# Upregulation of microRNA-4417 and Its Target Genes Contribute to Nickel Chloride-promoted Lung Epithelial Cell Fibrogenesis and Tumorigenesis

**DOI:** 10.1038/s41598-017-14610-7

**Published:** 2017-11-10

**Authors:** Chih-Hsien Wu, Yi-Min Hsiao, Kun-Tu Yeh, Tsui-Chun Tsou, Chih-Yi Chen, Ming-Fang Wu, Jiunn-Liang Ko

**Affiliations:** 1 Institute of Medicine, Chung Shan Medical University, Taichung, Taiwan; 2 Department of Medical Laboratory Science and Biotechnology, Central Taiwan University of Science and Technology, Taichung, Taiwan; 3 Department of Surgical Pathology, Changhua Christian Hospital, Changhua, Taiwan; 4 Division of Environmental Health and Occupational Medicine, National Health Research Institutes, Zhunan, Miaoli, 350 Taiwan; 5 Divisions of Medical Oncology and Pulmonary Medicine, Department of Internal Medicine, Chung Shan Medical University Hospital, Taichung, Taiwan; 6 School of Medicine, Chung Shan Medical University, Taichung, Taiwan; 7 Department of Medical Oncology and Chest Medicine, Chung Shan Medical University Hospital, Taichung, Taiwan; 8 Basic Medical Education Center, Central Taiwan University of Science and Technology, Taichung, Taiwan

## Abstract

Nickel compounds have been classified as carcinogens and shown to be associated with induction of epithelial-mesenchymal transition (EMT) in fibrogenesis and tumorigenesis, as well as the crucial role of microRNAs (miRNAs) and their related genes in controlling EMT and cancer metastasis. Thus, the mechanisms involved in the regulation of EMT in nickel-treated cells are of potential interest in understanding lung fibrosis and tumor progression. We investigated the miRNA-dependent mechanisms involved in nickel-induced EMT in lung epithelial cells. Nickel increased miR-4417 expression and decreased its target gene TAB2 expression. Treatment of cells with TGF-β inhibitor SB525334 significantly blocked NiCl_2_ and TGF-β-induced EMT. The expression of miR-4417 was abolished by SB525334 in TGF-β-treated cells, but not in nickel-treated cells. Both overexpression of miR-4417 and silencing of TAB2 induced fibronectin expression, but did not reduce E-cadherin expression. Moreover, oral administration of nickel promoted lung tumor growth in nude mice that had received BEAS-2B transformed cells by intravenous injection. The induction of EMT by nickel is mediated through multiple pathways. Induction of abundant miR-4417 and reduction of TAB2 expression following nickel exposure and may be involved in nickel-induced fibronectin. These findings provide novel insight into the roles of nickel in fibrogenesis and tumor progression.

## Introduction

In the past few years, evidence has indicated that genetic mutations and epigenetic changes are key drivers of epithelial-mesenchymal transition (EMT) in cancer cells. Recent studies have shed light on epigenetic modifications in cancer cells that cause a switch from non-invasive lesion to metastasis. Our previous study indicated that induction of EMT in lung bronchial epithelial cell line by nickel compounds is through various pathways. In addition, down-regulation of E-cadherin correlates with aberrant promoter methylation via ROS production in nickel-treated cells^1^. EMT-related epigenetic regulation of gene expression includes DNA methylation, histone modification, chromatin remodeling and small noncoding RNAs such as microRNAs (miRNAs)^2^.

miRNAs negatively regulate the stability and translation of target messenger RNAs (mRNAs) at the 3′-untranslated region (3′UTR)^3^. They have been reported to regulate the expression of >60% of human protein-coding genes^4^ and to be found in abundance in many human cell types^5^. Furthermore, miRNAs are aberrantly expressed in several types of cancer and involved in diverse processes, such as cell proliferation, control cell proliferation, differentiation, apoptosis, cell metastasis, angiogenesis, inflammation and carcinogenesis^6^. Down-regulation of miR-152 in NiS-transformed cells increases DNA methyltransferase 1 (DNMT1) and leads to a significant decrease in cell growth^7^. In an animal model, the expression of miR-222 significantly increased in nickel-induced tumor group when compared with normal group. In another study, miR-222 promoted cell proliferation during nickel-induced tumorigenesis in part by regulating the expression levels of its target genes, CDKN1B and CDKN1C^8^. Identification of oncogenic or tumoral suppressive miRNAs allows for their use as diagnostic and prognostic biomarkers and as potential targets in cancer therapies^9^.

EMT is a process by which the epithelial cells convert to a mesenchymal state that involves normal embryological development and an increase in fibroid morphology. It is implicated in various adult pathologies including cancer aggressiveness and metastasis^10^. EMT is regulated by a variety of signaling pathways that originate from the stromal cells and the surrounding microenvironment^11^. A critical molecular feature of EMT is repression of epithelial markers (e.g. E-cadherin) and induction of mesenchymal markers (e.g. fibronectin)^12^. miRNAs have been demonstrated to play important regulatory roles in EMT and may function as tumor promoters (oncomirs) or tumor suppressors (anti-oncomirs) depending on the miRNA and tumor type^13^. It has been demonstrated that miR-200 members inhibit EMT by targeting the E-cadherin repressors ZEB1 and ZEB2^14^. The identification of miRNA dysregulation in signaling pathways that induce EMT in oncogenesis has profound implications for the development of miRNA-based therapies.

Fibronectin, an extracellular matrix glycoprotein, is present on cell surfaces, and in extracellular fluids, connective tissues and basement membranes. Fibronectin plays a major role in development and wound healing by promoting cell adhesion, cytoskeletal organization and cancer cell metastasis^15^. Accumulation of fibronectin is an important pathological finding in fibrotic disorders^16^.

Epigenetic study of nickel-induced EMT by Wu, *et al*. revealed hypermethylation of the promoter of E-cadherin. However, very few studies have evaluated the effect of miRNAs on mesenchymal markers in nickel-induced EMT. Mesenchymal markers are fundamentally important to epigenetic regulation, but have not yet been studied in tumorigenesis.

In the present study, using miRNA and gene microarrays, we explored the possible regulatory role of a novel miR-4417 and further researched the mechanism involved in nickel-induced EMT. This is the first report to integrate miRNA expression data with mRNA expression data of cells treated with NiCl_2_ in the study of lung fibrogenesis and carcinogenesis.

## Results

### Nickel induces EMT partly via TGF-β signals

**Figure 1:**
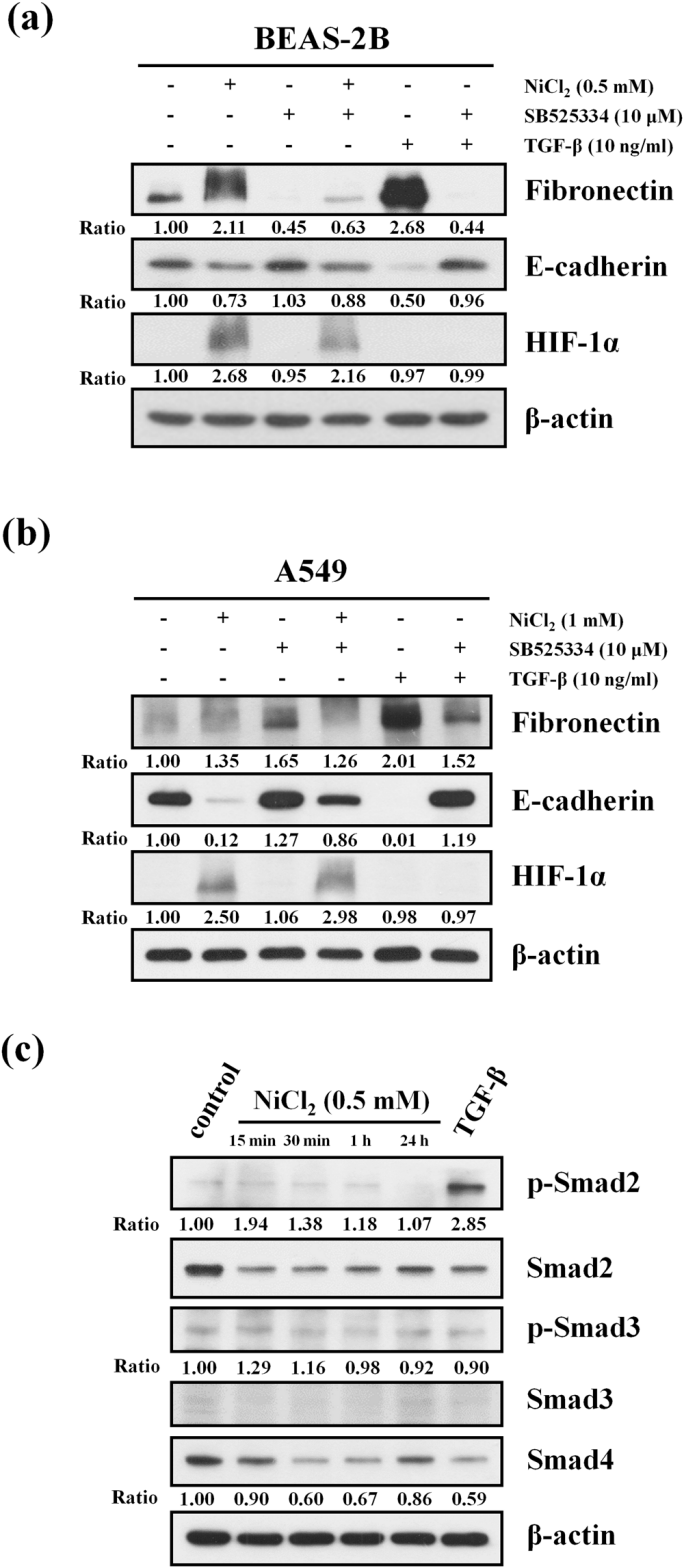
Effects of TGF-β receptor inhibition on EMT markers in nickel-treated cells. (**a**) BEAS-2B (1 × 10^6^ cells/6 cm dish) and (**b**) A549 (5 × 10^5^ cells/6 cm dish) cells were treated with NiCl_2_ (0.5 and 1 mM, respectively) or TGF-β (10 ng/ml) with or without SB525334 (10 μM) for 72 h and the protein levels were analyzed on Western blot. β-actin was used as the internal control. The relative ratios of Fibronectin/β-actin, E-cadherin/β-actin, HIF-1α/β-actin, TAB2/β-actin, ENOSF1/β-actin, and RAB6A/β-actin are shown. (**c**) BEAS-2B cells (1 × 10^6^ cells/6 cm dish) were treated with NiCl_2_ (0.5 mM) for 15 min, 30 min, 1 h and 24 h or TGF-β (10 ng/ml, positive control) for 30 min. The protein levels were analyzed on Western blot. β-actin was used as the internal control. The relative ratios of p-Smad2/Smad2, p-Smad3/Smad3 and Smad4/β-actin are shown.

In a previous study, NiCl_2_ induced EMT phenotype marker alterations such as up-regulation of fibronectin and down-regulation of E-cadherin^1^. To better understand the role of nickel compounds in tumor progression, we further explored the mechanism of tumorigenesis in immortalized human bronchial epithelial cell line (BEAS-2B cells) and lung cancer cell line (A549 cells). TGF-β signaling has been shown to be a potent driver of cancer progression through the induction of EMT, which enhances cell motility and metastasis^17^. To investigate whether NiCl_2_ induces EMT in lung carcinogenesis and fibrosis via TGF-β signaling pathway, we analyzed the effects of SB525334, a potent and selective inhibitor of the TGF-β receptor (activin receptor-like kinase, ALK5), on E-cadherin and fibronectin expressions in BEAS-2B and A549 cells. SB525334 was able to partially diminish up-regulation of fibronectin and restore E-cadherin expression in the presence of NiCl_2_. In the positive control (cultures treated with TGF-β), treatment with SB525334 resulted in abolished TGF-β-induced EMT (Fig. 1a and b). The expression of HIF-1α was upregulated by NiCl_2_. TGF-β did not increase HIF-1α protein levels.

It is well understood that TGF-β, through both Smad-dependent and Smad-independent mechanisms, plays a critical role in promoting cancer invasiveness and metastasis^18^. As shown in Fig. 1c, NiCl_2_ did not alter the p-smad2 expression, indicating that NiCl_2_ induces EMT via TGF-β/Smad-independent pathway and that more extensive communication occurs during signaling.

### miRNA microarray data analysis of NiCl_2_-treated cells

**Figure 2:**
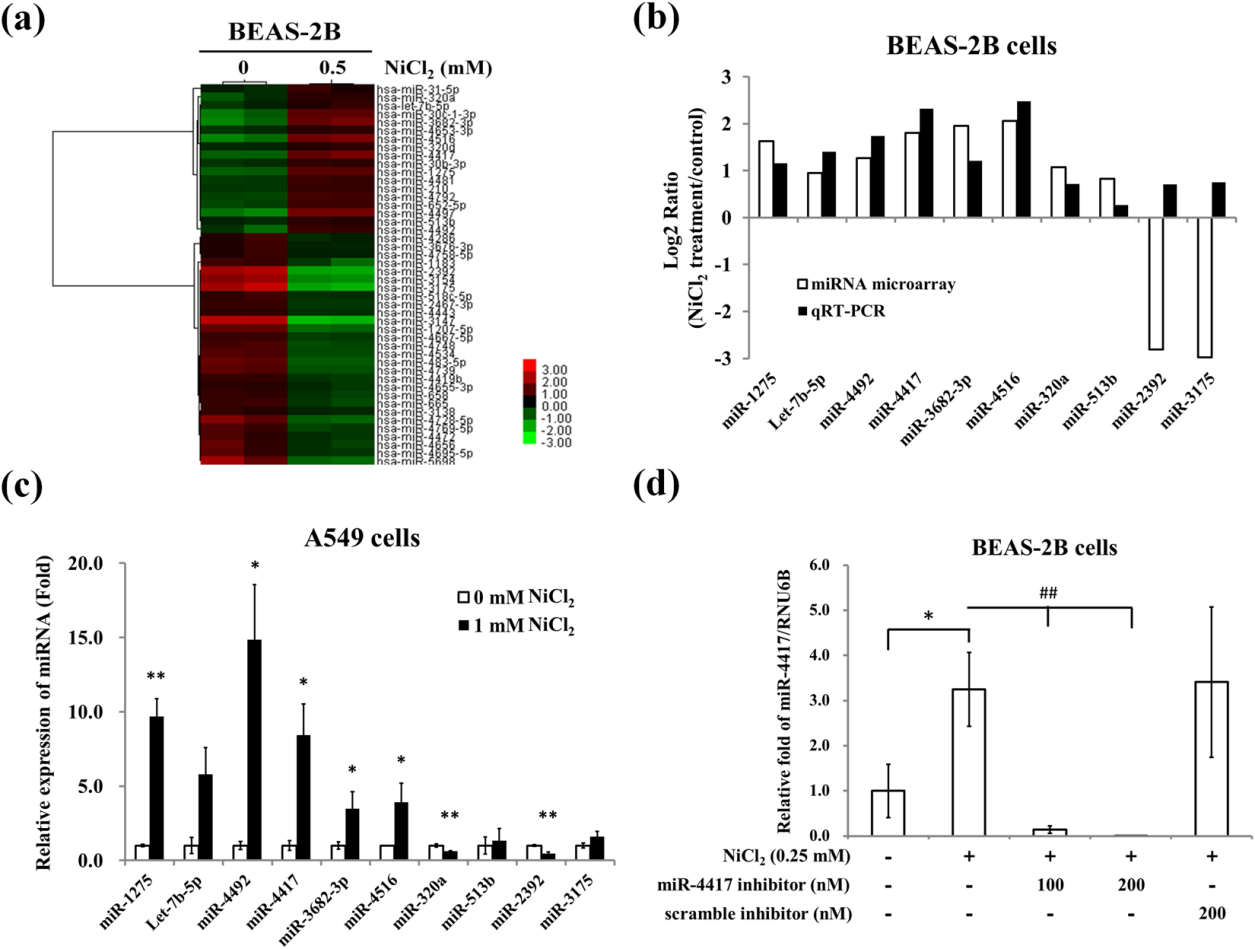
NiCl_2_ correlates with miRNA expression patterns. (**a**) BEAS-2B cells (1 × 10^6^ cells/6 cm dish) were treated with NiCl_2_ (0.5 mM) for 48 h. Heat maps and cluster analysis of the 46 miRNA differentially expressed between control and 0.5 mM NiCl_2_-treated BEAS-2B cells. Up- and down-regulated genes are represented in red and green, respectively. (**b**) Ten of the 46 miRNAs identified on microarray were corroborated with qRT-PCR data. BEAS-2B cells were treated with NiCl_2_ (0, 0.5 mM) for 48 h. The log2 of the ratios of expression levels are shown. (**c**) Seven miRNAs identified as significantly different between Ni-treated A549 cells and controls on microarray study were evaluated on qRT-PCR. All values have been normalized to the level of RNU6B and are the averages of three independent readings. *p < 0.05 and **P < 0.01 (**d**) BEAS-2B cells (1 × 10^6^ cells/6 cm dish) were transfected with microOFFTM miR-4417 inhibitor (100 and 200 nM) or scrambled miRNA inhibitor (scramble inhibitor, 200 nM) followed by exposure to NiCl_2_ (0 and 0.25 mM) for 48 h. Quantitative real-time PCR analysis was used to detect miR-4417 expression. All values have been normalized to the level of RNU6B and are the averages of three independent readings. *p < 0.05; ^##^p < 0.01.

To determine whether miRNA expression changes in human lung epithelial cells exposed to NiCl_2_, in comparison with untreated cells, total RNAs were obtained from BEAS-2B cells and analyzed on miRNA microarray. We identified 46 miRNAs differentially expressed between nickel-treated and untreated cells. Based on microarray threshold, there were 18 up-regulated and 28 down-regulated miRNAs with fold changes of ≥ 1.74 (log2 ratio ≥ 0.8) (Fig. 2a). To validate the microarray data, 10 miRNAs were randomly selected and real-time PCR was performed as an independent measure of miRNA expression. Of these 10 miRNAs, miR-1275, Let-7b-5p, miR-4492, miR-4417, miR-3682-3p, miR-4516, miR-320a and miR-513b were up-regulated, while miR-2392 and miR-3175 were down-regulated, by NiCl_2_, when compared with control group. All of these miRNAs were up-regulated by NiCl_2_ in BEAS-2B (Fig. 2b) and A549 cells (Fig. 2c). As miR-4417 was the most NiCl_2_-sensitive miRNA in BEAS-2B and A549 cells, we studied its functional significance using a specific inhibitor, miR-4417 inhibitor. NiCl_2_-induced expression of miR-4417 was abolished by transfection with miR-4417 inhibitor (100 and 200 nM) in BEAS-2B cells, while scrambled inhibitor had no observable effect (Fig. 2d).

### Analysis of miR-4417 targets in NiCl_2_-treated cells

**Figure 3:**
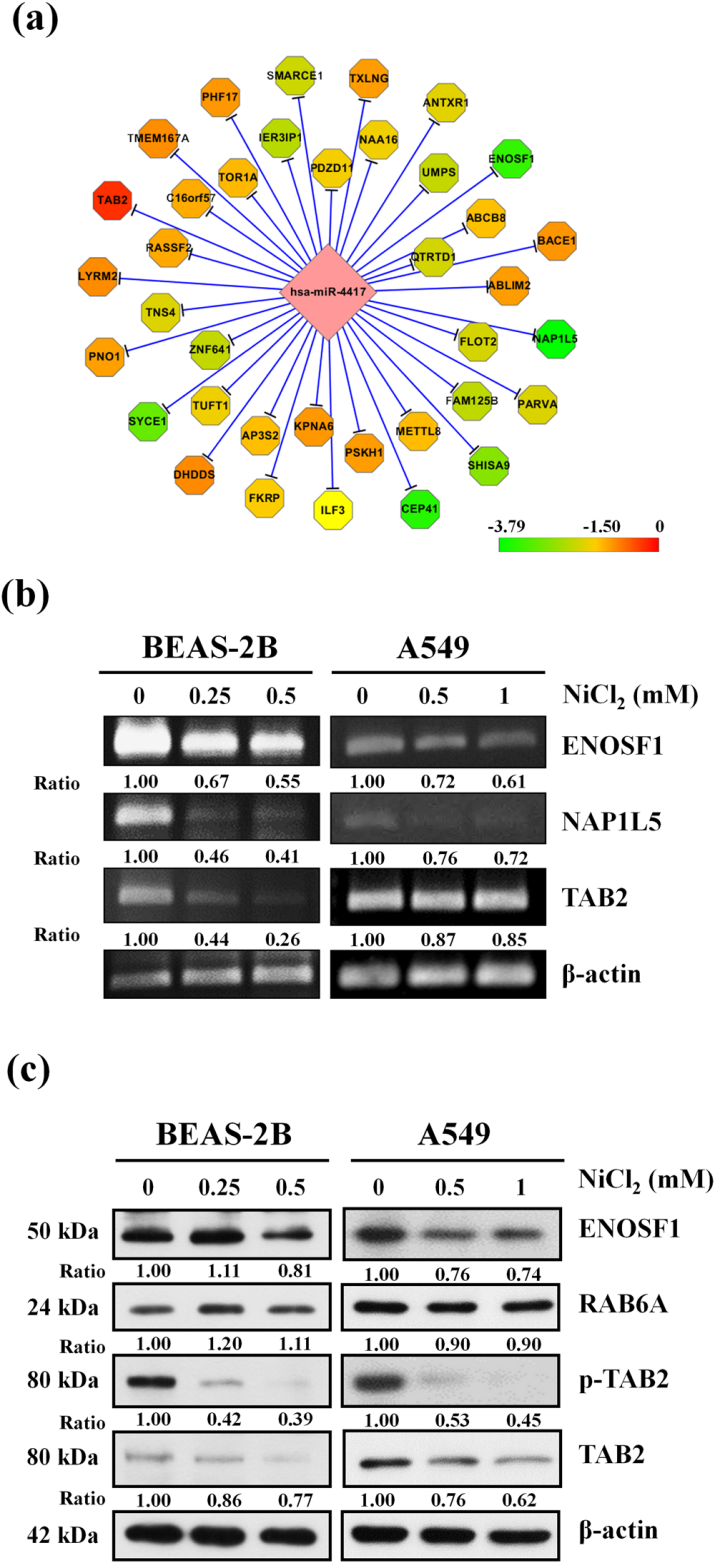
Network of miRNA target genes in nickel-treated cells. (**a**) Octagonal shapes represent mRNAs and diamond shape represents miR-4417. The figure was generated using CytoScape®. (**b**) BEAS-2B (1 × 10^6^ cells/6 cm dish) and A549 (5 × 10^5^ cells/6 cm dish) cells were treated with NiCl_2_ (0, 0.25, 0.5 mM and 0, 0.5, 1 mM, respectively) for 48 h. The mRNA levels were analyzed on RT-PCR. β-actin was used as the internal control. The relative ratios of ENOSF1/β-actin, NAP1L5/β-actin and TAB2/β-actin are shown. (**c**) BEAS-2B (1 × 10^6^ cells/6 cm dish) and A549 (5 × 10^5^ cells/6 cm dish) cells were treated with NiCl_2_ (0, 0.25, 0.5 mM and 0, 0.5, 1 mM, respectively) for 72 h and the protein levels were determined on Western blot. β-actin was used as the internal control. The relative ratios of ENOSF1/β-actin, RAB6A/β-actin, p-TAB2/β-actin and TAB2/β-actin are shown.

We performed gene expression microarray analysis using RNA isolated from BEAS-2B cells treated with NiCl_2_. Gene ontology (GO), MetaCore and Kyto Ecyclopedia Genes and Genomes (KEGG) were used to analyze the related pathways and networks of differentially expressed miR-4417 and its target mRNAs (Fig. 3a). To further evaluate whether the expressions of mRNA targets of miR-4417 are regulated by NiCl_2_, we analyzed gene expressions using RT-PCR. The treatment of BEAS-2B and A549 cells with various concentrations of NiCl_2_ led to the down-regulation of the gene expressions of ENOSF1, NAP1L5 and TAB2 (Fig. 3b). On Western blot, protein levels of ENOSF1, p-TAB2 and TAB2 decreased. However, RAB6A was not affected (Fig. 3c). These data suggest that NiCl_2_ down-regulates ENOSF1 and TAB2 via miR-4417.

### Nickel and TGF-β induce miR-4417 through different pathways

**Figure 4:**
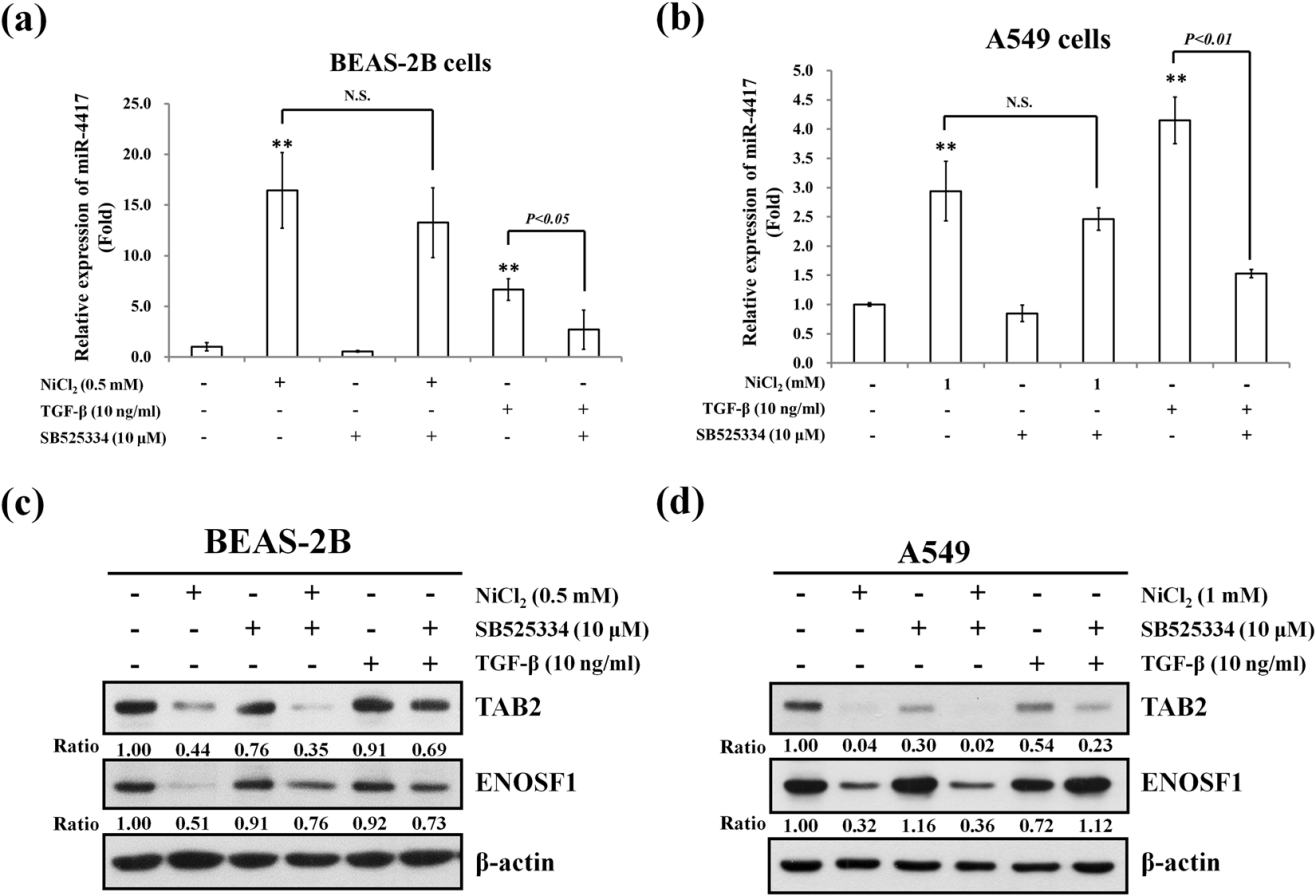
Effects of TGF-β inhibitor on miR-4417 target mRNAs in nickel-treated cells. (**a**) BEAS-2B and (**b**) A549 cells were treated with NiCl_2_ with or without SB525334 for 48 h and miR-4417 was analyzed on qRT-PCR. TGF-β-treated cells were used as the positive control.*p < 0.05, **p < 0.01 (compared with untreated control) and N.S. (non-significance) (**c**) BEAS-2B (1 × 10^6^ cells/6 cm dish) and (**d**) A549 (5 × 10^5^ cells/6 cm dish) cells were treated with NiCl_2_ (0.5 and 1 mM, respectively) or TGF-β (10 ng/ml) with or without SB525334 (10 μM) for 72 h and the protein levels were analyzed on Western blot. β-actin was used as the internal control. The relative ratios of TAB2/β-actin and ENOSF1/β-actin are shown.

As shown in Fig. 1a and b, TGF-β was involved in nickel-induced EMT based on molecular marker analysis of gene expression profiles. To explore novel potential regulators of EMT, we investigated whether TGF-β participates in nickel-induced EMT via miR-4417. In response to treatment with SB525334, TGF-β-induced miR-4417 was significantly inhibited in BEAS-2B and A549 cells. However, SB525334 only partially reduced nickel-induced expression of miR-4417, with no significant differences between groups (Fig. 4a and b). Next, we examined whether TGF-β signaling is associated with the expressions of miR-4417 target genes down-regulated by NiCl_2_. Treatment with SB525334 did not reverse the protein levels of TAB2 or ENOSF1 in NiCl_2_-treated cells. Stimulation of BEAS-2B and A549 cells with TGF-β resulted in no change in TAB2 or ENOSF1 (Fig. 4c and d). These data indicated that NiCl_2_ and TGF-β induce miR-4417 and its target genes through different signaling pathways.

### Nickel induces fibronectin by down-regulation of TAB2 via miR-4417

**Figure 5:**
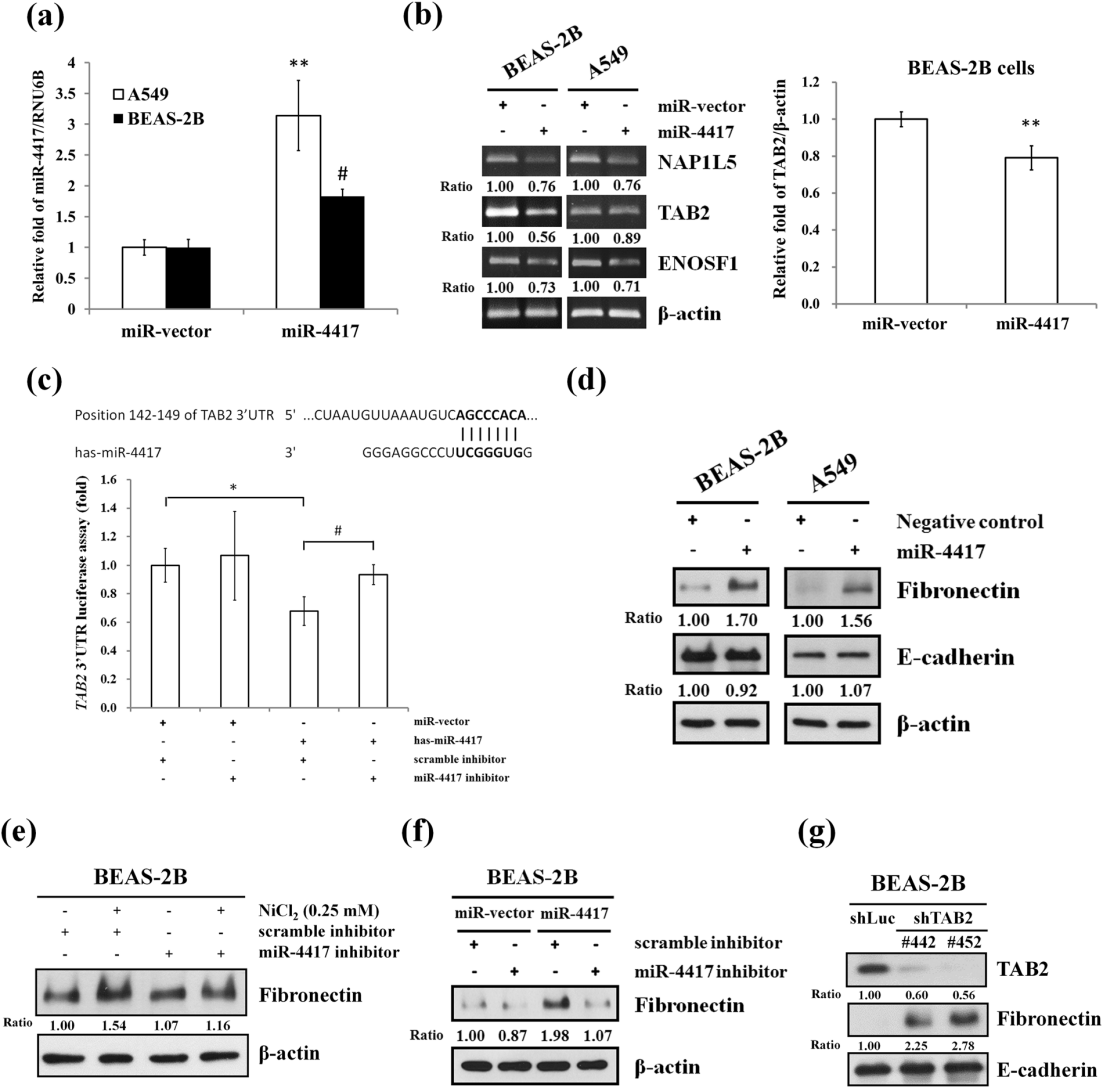
Effects of miR-4417 on mesenchymal marker fibronection. (**a**) A549 and BEAS-2B cells were transfected with miR-vector (vector control) or miR-4417/pcDNA^TM^ 6.2-GW for 48 h. Quantitative real-time PCR analysis was used to detect miR-4417 expression. All values are normalized to the level of RNU6B. Each bar represents the mean ± SD of triplicate experiments; ^#^p < 0.05 (compared to BEAS-2B miR-vector cells) and **p < 0.01 (compared to A549 miR-vector cells). (**b**) Left, the mRNA levels of miR-4417-targeted genes, including NAP1L5 and TAB2, were determined on RT-PCR. β-actin was used as the internal control. The relative ratios of NAP1L5/β-actin and TAB2/β-actin are shown. Right, the TAB2 mRNA levels were determined on real-time PCR. **p < 0.01 (**c**) Top, the predicted hsa-miR-4417 seed sequence binding site of the TAB2 3′UTR using miRDB. Bottom, BEAS-2B cells were cotransfected with psiCHECK-2/TAB2 3′UTR and miR-vector or hsa-miR-4417 with scramble inhibitor or miR-4417 inhibitor plasmid. After 48 h, the transcriptional activity of each reporter plasmid was normalized relative to β-galactosidase activity. Each bar represents the mean ± SD of triplicate experiments; *and ^#^p < 0.01 (**d**) BEAS-2B and A549 cells were transfected with negative control or miR-4417 mimic (100 nM) for 48 h, and analyzed by Western blot. β-actin was used as the internal control. The relative ratios of Fibronectin/β-actin and E-cadherin/β-actin are shown. (**e**) BEAS-2B cells were transfected with or without microOFF^TM^ miR-4417 inhibitor (100 nM) or scrambled miRNA inhibitor (scrambled inhibitor, 100 nM) followed by exposure to NiCl_2_ (0 and 0.25 mM) for 48 h. (**f**) BEAS-2B cells were co-transfected with miR-4417/pcDNA^TM^ 6.2-GW (5 μg) and miR-4417 inhibitor (100 nM) or scrambled miRNA inhibitor (scrambled inhibitor, 100 nM), and analyzed by Western blot. (**g**) BEAS-2B cells were infected with lentivirus carrying shTAB2 #442 or #452 or vector control (shLuc) and analyzed by Western blot. β-actin was used as the internal control. The relative ratios of TAB2/β-actin, Fibronectin/β-actin and E-cadherin/β-actin are shown.

We next investigated the role of miR-4417 in NiCl_2_-induced EMT. BEAS-2B and A549 cells were transfected with plasmid that expresses hsa-miR-4417 (miR-4417) or miRNA scrambled control vector (miR-vector). Quantitative real-time PCR showed that the expressions of miR-4417 are up-regulated in the miR-4417 groups of BEAS-2B and A549 cells (Fig. 5a). To test the regulatory functions of exogenous pre-miR-4417, we analyzed the expressions of miR-4417-targeted mRNAs on RT-PCR. Overexpression of miR-4417 led to a corresponding decrease in endogenous NAP1L5, TAB2 and ENOSF1 mRNAs (Fig. 5b). The miR-4417 target sites in TAB2 3′-untranslated region (UTR) were predicted using miRDB (http://mirdb.org/miRDB/)^19^. Luciferase activity was significantly decreased in co-transfected with hsa-miR-4417 and psiCHECK-2/TAB2 3′UTR plasmid cells compared with its activity in the miR-vector and miR-4417 inhibitor-treated cells. miR-4417 inhibitor was reversed the luciferase activity of TAB2 3′UTR in BEAS-2B transfected-miR-4417 cells (Fig. 5c). Together, these data demonstrated that TAB2 is a target gene of miR-4417 in BEAS-2B cells. Interestingly, we found that overexpression of miR-4417 enhanced the protein levels of fibronectin, but had no obvious inhibitory effect on E-cadherin (Fig. 5d). Transfection of BEAS-2B cells with miR-4417 inhibitor repressed NiCl_2_ and miR-4417-induced fibronectin levels when compared with scrambled inhibitor group (Fig. 5e and f), indicating that miR-4417 is involved in nickel-mediated mesenchymal characteristics. To clarify the mechanism of fibronectin up-regulation by NiCl_2_, we stably silenced miR-4417-targeted gene TAB2 with lentiviral short hairpin RNA. The responses of BEAS-2B shLuc cells were similar to those of parental BEAS-2B cells. Silencing of TAB2 increased fibronectin expression, but had no effect on E-cadherin expression (Fig. 5g). These results suggested that upregulation of miR-4417 and downregulation of TAB2 are associated with nickel-induced the expression of fibronectin.

### Nickel accumulates in organs and induces lung carcinogenesis

One of the aims of this study was to determine the distribution of nickel to better understand the relationship between nickel exposure and levels of nickel accumulation in the organs of mice. Eight-week-old female immunodeficient nude mice, pretreated for 15 days with NiCl_2_ (0, 20 and 100 mg/kg/day), were intravenously (iv) injected with BEAS-2B cells. They were subsequently administered NiCl_2_ at 0, 20 or 100 mg/kg/day by oral gavage for 60 days and sacrificed on day 75. Brain, liver, lung, stomach, spleen and kidney were collected for nickel analysis on ICP-MS (Agilent 7700X). In the group exposed to NiCl_2_ at 20 mg/kg/day, the mean concentrations of nickel in lung (550.51 ± 130.25 ppb) and liver (130.95 ± 3.29 ppb) were significantly higher than in control group (86.60 ± 70.68 and 27.49 ± 13.92 ppb, respectively). In the group exposed to NiCl_2_ at 100 mg/kg/day, the mean concentrations of nickel in lung (5739.75 ± 809.61 ppb), kidney (5615.81 ± 1581.52 ppb), liver (779.42 ± 45.79 ppb), brain (798.12 ± 67.63 ppb) and spleen (2920.64 ± 624.81 ppb) were significantly higher than in control group (86.60 ± 70.68, 60.13 ± 28.09, 27.49 ± 13.92, 43.37 ± 35.02 and 203.09 ± 93.27 ppb, respectively) (Table 1).

**Table 1:** The contents of nickel in each organ of mice 75 days exposure to different dose nickel chloride by oral administration at of 20 or 40 mg/kg/day.

Ni (ppb)	control group	Ni-exposed group	
	ddH_2_O	20 mg/kg/day	100 mg/kg/day
lung	86.60 ± 70.68	550.51 ± 130.25^*^	5739.75 ± 809.61^*^
kidney	60.13 ± 28.09	1298.21 ± 602.40	5615.81 ± 1581.52^*^
liver	27.49 ± 13.92	130.95 ± 3.29^**^	779.42 ± 45.79^**^
brain	43.37 ± 35.02	127.40 ± 21.24	798.12 ± 67.63^*^
spleen	203.09 ± 93.27	424.29 ± 47.36	2920.64 ± 624.81^*^
stomach	94.34 ± 29.22	296.92 ± 182.90	27567.55 ± 27787.24

Note: Values represent mean ± SEM.

^*^p < 0.05 and ^**^p < 0.01^,^ significantly different from control group.

**Figure 6:**
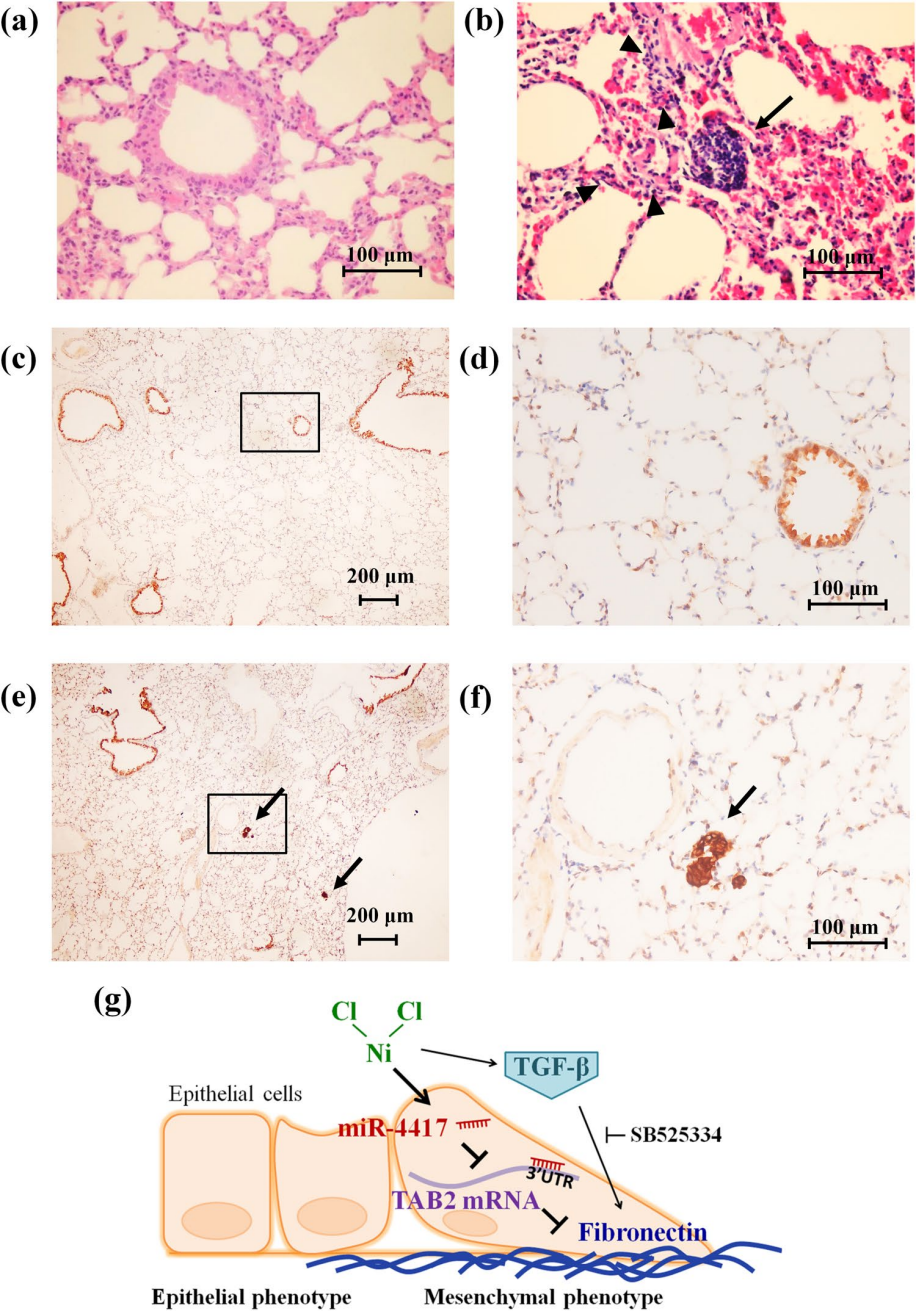
Photomicrographs of representative lung cancer formation in nickel-treated mice. After oral administration of 0 and 20 mg NiCl_2_/kg/day for 15 days, the mice were inoculated with or without BEAS-2B cells (1.5 × 10^6^ cells/mouse) via intravenous route. The mice were continually exposed to (**a**) ddH_2_O or (**b**) NiCl_2_ for 60 days and sacrificed at 75 days. The tumor development in lung tissues were investigated by H&E stain. Photographed at 100× magnification. Black arrow points to the tumor cells and black triangles to cell fibrogenesis. Lung tissue sections from ddH_2_O group (**c** and **d**) and NiCl_2_ group (**e** and **f**) were analyzed by immunohistochemistry using a human cytokeratin 19 antibody. The compositional range enclosed by the quadrilateral of (**c**) and (**e**) are enlarged in (**d**) and (**f**), respectively. Photomicrographs of IHC for human cytokeratin 19 in the lung tissue of the nude (magnification: left, 40× and right, 100×). The black arrows point to the tumor cells. (**g**) Model depicting the mechanisms of Ni-induced fibronectin.

To verify the malignant transformation effect of NiCl_2_ on immortalized human cells, we used H&E stain to analyze tumor formation. Here we found that repetitve exposure to NiCl_2_ (20 mg/kg) promoted lung cell proliferation and lung tumorigenesis (Fig. 6a and b). Exposure to NiCl_2_ (100 mg/kg) induced fatty liver. There were no obvious pathologic changes in the kidney of NiCl_2_-treated groups when compared with the control group (SFig. 1a–d). To determine whether NiCl_2_ promotes BEAS-2B cells tumorigenesis in mouse lung, human cytokeratin 19 were identify by using IHC. High level of human cytokeratin 19 was found in the tumor sections in nickel-treated group compared with that of control group (Fig. 6c–f).

The tracheal muscle was stained with Masson’s trichrome in the both ddH_2_O and NiCl_2_ group mice (black arrow, SFig. 2a–d). Nickel administration resulted in gradually exacerbated lung alveolar destruction, interstitium thickening and fibroblast diffusion in the NiCl_2_ group mice (black triangles, SFig. 2c and d). In these results, we found that nickel accumulation in lung induce tumorigenesis and fibrogenesis.

## Discussion

Metastasis is a multistep process that involves the spread of cancer cells from a primary tumor to seed secondary tumors in distant places, which is common in late stage disease and correlated with poor prognosis^20^. EMT, an important developmental program in motility and dissemination of cancer cells, is dynamically and reversibly regulated during transdifferentiation^21^. Gastrointestinal distress (e.g. nausea, vomiting, abdominal pain, diarrhea), neurological effects (e.g. headache), cough, shortness of breath and giddiness were reported in workers at an electroplating plant who drank water contaminated with NiCl_2_ and nickel sulfate (NiSO_4_)^22^. In 1990, the International Agency for Research on Cancer (IARC) classified Ni (II) compounds as known carcinogenic to humans (group 1)^23^. Animal studies have indicated that toxic effects of oral exposure to nickel-soluble salts involve the kidneys with some evidence of developmental/reproductive toxicity effects^24^. Although several studies have been conducted to assess the carcinogenic potential of nickel compounds following oral exposure, animal experiments have suffered from inadequate design. In this study, we first investigated whether oral exposure of NiCl_2_ promotes disseminated (metastatic) tumors in nude mice produced by intravenous injection of BEAS-2B cells. In BEAS-2B cells, NiCl_2_ exposure induced malignant transformation accompanied by EMT. These findings imply a novel role for nickel compounds in lung cancer formation and progression.

Epidemiological studies have shown that the chronic occupational exposure to nickel compounds via inhalation, ingestion or percutaneously in the workplace increases the risk of respiratory cancers^25,26^. Nickel exposure has been associated with increased the risk of p53 mutation in lung cancer due to decrease DNA repair capability^27^. Furthermore, recent research indicate that nickel induce miR-21 via activation of the EGFR/NF-κB pathway and consequently promote tumor invasion. Accumulation of nickel in lung tissue contribute to EGFR mutation via upregulating miR-21 in never-smoking patients with lung cancer^28^. These studies strongly suggest that nickel not only induce tumor initiation but also accelerated migration of human lung cancer cells.

Activation of Toll-like receptor 4 (TLR4) signaling by nickel can trigger both the MyD88-dependent and MyD88-independent pathway^29^. The TLR4 can induce the activation of NF-κB pathway which is conferred the enhanced invasion of lung cancer cells induced by nickel^30,31^. Nickel chloride promotes the invasive potential of human lung cancer cells through elevated IL-8, TGF-β, MMP2 and MMP9 expression^31^. In a murine model of organ fibrosis and EMT induced by TGF-β can be inhibited by Bone morphogenetic protein-7 (BMP-7)^32^. Thus, BMP-7 may have the potential for nickel-induced lung fibrosis.

TLR4 play a dominant role in NF-κB activation in nickel-induced inflammatory response^33^. Stimulation of Toll-like receptors (TLRs) triggers the association of myeloid differentiation primary-response protein 88 (MyD88) thereby allowing the activation of protein kinase TGF-β-activated kinase 1 (TAK1) by binding to Lys63 (K63)-linked polyubiquitin chains through adaptor protein, including TAB2 and TAB3, and in turn induces transcription factor NF-κB activation^34,35^. In this study, the RNA and protein levels of TAB2 expression are decreased in nickel-treated cells. Ubiquitin (Ub) is well known as a signal that targets protein substrates for degradation by the 26S proteasome. Therefore, nickel might induce ubiquitination of TAB2 and IκB resulted in TAB2 and IκB degradation for NF-κB activation. However, overexpression of miR-4417 did not inhibit the protein levels of TAB2 (data not shown). We speculate that exogenous miR-4417 would not trigger TLR4 activation and do not signal for the target TAB2 ubiquitination and degratdation.

The development of lung fibrosis is one of the early steps in the pathogenesis of advanced restrictive lung diseases and respiratory failure. Fibronectin is a ubiquitous extracellular glycoprotein that exists in both a soluble form in plasma and an insoluble form in the extracellular matrix (ECM)^36^. Recent studies have suggested that TGF-β is a major stimulator of ECM protein production and hence fibronectin expression through Smad-dependent pathway and mitogen-activated protein kinase (MAPK) pathway^37,38^. NiCl_2_ induces EMT phenotype marker alteration, including up-regulation of fibronectin^1^. Together, these results support nickel-induced EMT via various other pathways.

Previously, TAB1 and TAB2 were isolated from TAK1-binding complex by yeast two-hybrid screening. TAB2 has been shown to be an adaptor linking TAK1 and TRAF6 in IL-1-induced NF-κB and MAPK pathway and to mediate TAK1 activation^39^. It has been demonstrated that IL-1 induces protein TAB2 interaction with estrogen receptor alpha (ERα)/nuclear receptor co-repressor (NCoR) and causes dismissal of NCoR from these genes, leading to the loss of the antiproliferation response^40^. In the present study, down-regulation of TAB2 increased fibronection expression, which is involved in nickel-induced EMT and can subsequently promote tumor metastasis. This points to a novel mechanism for the regulation of fibronectin. We therefore hypothesized that treatment of BEAS-2B cells with NiCl_2_ inhibits cell proliferation to induce cell transformation and metastasis. However, this hypothesis remains to be confirmed in further experiments.

Non-alcoholic fatty liver disease (NAFLD) progresses slowly and can develop into liver cirrhosis, liver failure, and primary liver cancer (hepatocellular carcinoma, HCC)^41^. The activities of hepatic marker enzymes (ALT and AST) in serum increase significantly following treatment with NiCl_2_ in normal rats in a dose-dependent manner. NiCl_2_-treated rats also show alterations in normal hepatic histoarchitecture with increased lipid peroxidation and development of microvesicular steatosis and fatty liver^42^. In this study, following oral administration of soluble nickel salts, nickel was found to accumulate in liver (Table 1) and promote fatty liver development (Fig. 6). However, we did not find more serious effects. A 75-day exposure period may not have been long enough to generate mice models of liver cirrhosis and liver cancer.

Most human cancers originate from epithelial cells such as cancers of the breast, lung, colon and skin, which collectively lead to several million deaths per year^43^. Tumor cells must acquire certain characteristics for cell invasion and migration via a process known as EMT^32^. Members of the TGF-β family have been identified as particularly efficient inducers of EMT. However, EMT is also promoted by a number of other factors, including Wnt, Notch, several tyrosine kinase ligands and nickel compounds, during development and carcinogenesis^1,17,44^. In this study, TGF-β and NiCl_2_ both induced miR-4417 expression, but TGF-β did not affect HIF-1α or TAB2 (Figs 1a,b, 2c,d). Our results indicated that nickel-induced EMT partially involves TGF-β-mediated smad-2 independent pathway.

In this study, we identified 8 nickel-sensitive miRNAs in cultured BEAS-2B cells by performing genome-wide miRNA microarray and subsequent validation on real-time PCR analysis (Fig. 2a and b). Then, we determined the functional importance of the most nickel-sensitive miRNA, miR-4417, in nickel-treated BEAS-2B and A549 cells. Using hsa-miR-4417 precursor and miR-4417-specific inhibitor, we found that miR-4417 specifically mediates nickel-induced fibronectin expression, but does not affect E-cadherin expression (Fig. 5d). We also performed additional genome-wide DNA microarray analysis to identify potential gene targets regulated by miR-4417 in nickel-treated cells. The results showed that 37 potential genes are regulated (Fig. 3a). Among them, TAB2 was down-regulated and fibronectin was upregulated by NiCl_2_, suggesting that up-regulation of miR-4417 and inhibition of TAB2 are related to nickel-induced EMT (Fig. 6g).

## Materials and Methods

### Cell culture

The following cell lines were obtained from ATCC (Manassas, VA): BEAS-2B and A549. BEAS-2B (an SV40-immortalized human bronchial epithelial cell line) cells were cultured in serum-free LHC-9 medium (Invitrogen, Carlsbad, CA). A549 (a human lung adenocarcinoma epithelial cell line) cells were cultured in Dulbecco’s modified Eagle’s medium (DMEM; Gibco, Invitrogen, Carlsbad, CA) containing 10% fetal bovine serum (FBS). The cells were grown in an incubator at 37 °C in a humidified atmosphere of 5% CO_2_. Cell lines were passaged and maintained for fewer 6 months after resuscitated and were routinely authenticated, including growth curve analysis, species verification by karyotyping and isoenzymology, cell morphology observing, identity verification using short tandem repeat profiling analysis, and contamination checks. The cells were last tested in Mar 2015.

NiCl_2_ was purchased from Sigma Chemical Company (N-6136, St. Louis, MO, USA). TGF-β receptor I inhibitor SB525334 was obtained from Selleck Chemicals (Houston, TX, USA). Recombinant human TGF-β was obtained from PeproTech (100-21 C, London, UK). miR-4417 inhibitors and scrambled inhibitors were purchased from Guangzhou RiboBio (RiboBio, Guangzhou, China). miRNA mimic of hsa-miR-4417 (ABGP020100) and negative control (ABGP020101) were from Allbio Science Inc. The working concentration of miRNA mimic was 100 nM. Precursor miRNA expression clone for hsa-miR-4417 (HmiR1076-MR04) and miRNA scrambled control clone (miR-vector) for pEZX-MR04 (CmiR0001-MR04) were purchased from GeneCopoeia^TM^ (Germantown, MD, USA) and transfected into cells using Lipofectamine 2000 (Invitrogen, Life Technologies, CA, USA) as recommended by the manufacturer.

### Western blot analysis

Cell lysates were collected in RIPA buffer. Proteins were separated on SDS-PAGE and subsequently transferred from gel onto PVDF membrane (Pall, Port Washington, NY). Membranes were blocked for 1 h at room temperature with 5% non-fat dry milk in Tris-buffered saline plus 0.01% Tween-20 (TBS-T). Smad2/3 antibody sampler kit (Cell Signaling, #12747), anti-fibronectin (BD, 610077), anti-E-cadherin (BD, 610182), anti-HIF-1α (BD, 610959), anti-ENOSF1 (GeneTex, GTX119464), anti-RAB6A (GeneTex, GTX110646), anti-phopho-TAB2 (Cell Signaling, #8155), anti-TAB2 (Cell Signaling, #3745) and anti-β-actin (Sigma, A5441) antibodies, were used to detect p-Smad2, Smad2, p-Smad3, Smad3, Smad4, fibronectin, E-cadherin, HIF-1α, ENOSF1, RAB6A, p-TAB2, TAB2 and β-actin, respectively, with incubation of membrane at 4 °C overnight. After washing three times with TBS-T, membranes were incubated with HRP-conjugated secondary antibodies at room temperature for 1 h. Signal was detected with an enhanced chemiluminescence detection kit (Perkin Elmer Life Sciences, Boston, MA).

### RNA extraction and microarray analysis

Total RNA from cell lines was extracted using RareRNA (Genepure Technology, Taiwan) following the manufacturer’s protocol. mRNA and miRNA profiles were established using Agilent SurePrint G3 Human V2 GE 480 K and HmiOA4.1 microarrays, respectively. Then, Gene ontology (GO), MetaCore and Kyto Ecyclopedia Genes and Genomes (KEGG) were used to analyze the related pathways and networks of differentially expressed miRNAs and mRNAs.

### Reverse transcriptase-polymerase chain reaction (RT-PCR)

Reverse transcription of 2 μg of total cellular RNA was performed in a final volume of 20 μl containing 10 µl RNA, 2 µl 10X RT buffer, 0.8 µl dNTP Mix (100 mM), 2.0 µl 10X RT random hexamer primers, 1.0 µl MultiScribe™ reverse transcriptase, 1 µl RNase inhibitor and 3.2 µl nuclease-free water (Applied Biosystems, Foster City, USA). The reverse transcription reaction conditions were 25 °C for 10 minutes, 37 °C for 120 minutes, and 85 °C for 5 seconds. cDNA samples were stored at −20 °C. Primer sequences and annealing temperatures were shown in Supplemental Table 1. RT-PCR products were visualized by agarose gel electrophoresis with ethidium bromide staining. The level of mRNA expression was normalized to that of β-actin mRNA.

### miRNA isolation and reverse transcription

miRNAs were extracted from cell lines using the High Pure miRNA Isolation Kit (Roche Diagnostic GmbH, Mannheim, Germany) according to the manufacturer’s instructions and stored at −80 °C for further processing. Reverse transcription was performed on 11 µl of total miRNA using Transcriptor Reverse Transcriptase (Roche), which consisted of 4 µl of 5X Transcriptor RT reaction buffer, 0.5 µl of Protector RNase inhibitor (40 U/µl), 2 µl of 10 mM dNTP-Mix and 0.5 µl of Transcriptor Reverse Transcriptase, with pre-incubation at 65 °C for 5 min. After cooling on ice for 2 min, the mixtures were incubated at 16 °C for 30 min, followed by 60 cycles at 30 °C for 30 s, 42 °C for 30 s and 50 °C for 1 s. Then, inactive Transcriptor Reverse Transcriptase was heated to 85 °C for 5 min and the tube was stored at −20 °C for further processing.

### Quantitative real-time PCR

Reverse transcription and real-time RT-PCR for miRNAs were performed using the Bulge-Loop miRNA real-time RT-PCR Primer Set (RiboBio, Guangzhou, China). The real-time PCR reactions were carried out with Smart Quant Green Master Mix with dUTP or Smart Quant Probe Master Mix with dUTP & ROX, 2x Mix (Protech Technology), and on 96-well microtiter plate using StepOne™ real-time PCR machine (Applied Biosystems, Foster City, USA). The PCR conditions were 95 °C for 15 min followed by 45 cycles at 94 °C for 15 s and 60 °C for 1 min with data acquisition after each cycle. The fluorescent signals were collected during extension phase. Ct values of the samples were calculated, and the transcript levels were analyzed by 2^−ΔΔCt^ method. For normalization of real-time PCR result in miRNA quantification, U6 small nuclear RNA (RNU6B, Assay no. 4427975; Applied Biosystems, USA) was used as an internal loading control.

### RNA interference

Cells were transfected with plasmid containing short hairpin RNAs (shRNAs) of human TAB2 obtained from the National RNAi Core Facility located at the Institute of Molecular Biology/Genomic Research Center, Academia Sinica, Taiwan. Individual clones were identified by their unique TRC number, e.g., shLuc TRCN0000072246 for vector control targeted to luciferase; shTAB2 (#442, TRCN0000378442, target sequence: GTGATGAAAGAATTACCGAAT) and shTAB2 (#452, TRCN0000004452, target sequence: AGATTGACATTGACTGCTTAA) for vector control targeted to TAB2. The cells were selected using 2 μg/ml puromycin (Sigma, P8833).

### Luciferase activity assay

The psiCHECK-2/TAB2 3′UTR plasmid was kindly provided by Dr. Isabelle Dunand-Sauthier (University of Geneva Medical School, Geneva, Switzerland). BEAS-2B cells were cultured in 12-well plate (3 × 10^5^ cells per well), co-transfected with 0.4 μg miR-vector (GeneCopoeia, CmiR0001-MR04) or hsa-miR-4417 (GeneCopoeia, HmiR1076-MR04), 0.4 μg psiCHECK-2/TAB2 3′UTR and 0.2 μg pCMV-β-galactosidase, and treated with 100 nM miR-4417 inhibitor or scramble inhibitor (RiboBio, Guangzhou, China) by lipofectamine 2000. After 48 h, cells were harvested using lysis buffer and luciferase activity was measured using the Luciferase Assay System (Promega).

### Statistical analysis

Statistical analysis was performed using the Predictive Analytics Software (PASW, SPSS Inc) V.18. Data are presented as mean ± SD. The comparisons between two groups were conducted using One-sample *t test*. *P* values of < 0.05 were considered significant.

### Xenograft tumor model

All animal experiments were conducted in accordance with the committee guidelines of the Chung Shan Medical University and approved by the IACUC (Institutional Animal Care and Use Committee of Chung Shan Medical University, Ethical NO. 839). Six-week-old female BALB/AnN.Cg-Foxnlnu/CrlNarl nude mice were purchased from the National Laboratory Animal Center (Taipei, Taiwan, R.O.C.). After 2 weeks, immunodeficient nude mice, pretreated for 15 days with NiCl_2_ (0, 20 and 100 mg/kg/day), were intravenously (iv) injected with BEAS-2B cells (1.5 × 10^6^ cells/mouse, n = 3/each group). Mice were subsequently administered NiCl_2_ (0, 20 or 100 mg/kg/day) by oral gavage for 60 days and sacrificed on day 75. Organs were fixed with 10% neutral formalin and embedded in paraffin. Sections were stained with hematoxylin and eosin (H&E) stain for histopathologic examination under light microscopy.

## Electronic supplementary material

Supplemental table and figures
