# Supplementary material for: Upregulation of microRNA-4417 and Its Target Genes Contribute to Nickel Chloride-promoted Lung Epithelial Cell Fibrogenesis and Tumorigenesis
Source: Sci Rep. 2017 Nov 10;7:15320. doi: 10.1038/s41598-017-14610-7 (PMC5681645; doi:10.1038/s41598-017-14610-7)
Supplement: Supplementary file 1 — Supplemental table and figures [file 41598_2017_14610_MOESM1_ESM.pdf]

Title

Upregulation of microRNA-4417 and Its Target Genes Contributes to Nickel Chloride-promoted Lung Epithelial Cell Fibrogenesis and Tumorigenesis

Author list and affiliations

Chih-Hsien Wu[1, 8], Yi-Min Hsiao[2], Kun-Tu Yeh[3, 6], Tsui-Chun Tsou[4], Chih-Yi Chen[1, 6], Ming-Fang Wu[5, 6, 7]*[†] and Jiunn-Liang Ko[1, 7]*[†]

[1]Institute of Medicine, Chung Shan Medical University, Taichung, Taiwan

[2]Department of Medical Laboratory Science and Biotechnology, Central Taiwan University of Science and Technology, Taichung, Taiwan

[3]Department of Surgical Pathology, Changhua Christian Hospital, Changhua, Taiwan

[4]Division of Environmental Health and Occupational Medicine, National Health Research Institutes, Zhunan, Miaoli 350, Taiwan

[5]Divisions of Medical Oncology and Pulmonary Medicine, Department of Internal Medicine, Chung Shan Medical University Hospital, Taichung, Taiwan

[6]School of Medicine, Chung Shan Medical University, Taichung, Taiwan

[7]Department of Medical Oncology and Chest Medicine, Chung Shan Medical University Hospital, Taichung, Taiwan

[8]Basic Medical Education Center, Central Taiwan University of Science and Technology, Taichung, Taiwan

*****Correspondence and request for materials should be addressed to J.-L. Ko or M.-F. Wu (E-mail: jlko@csmu.edu.tw or mfwu0111@gmail.com) Institute of Medicine, Chung Shan Medical University, 110, Sec. 1, Chien-Kuo N. Road, Taichung, Taiwan 40201 Tel: (886-4) 24730022-11694; Fax: (886-4) 24751101

[†] These authors contributed equally to this work.

Supplemental Table 1. List of primer sequences (5'-3') for RT-PCR

Genes	Forward primer	Reverse primer	Annealing temperature (℃)	product size (bp)
ENOS1	CATGCGAAGATGCCAAATCATCC	CAACTGTCAATCTGGAGGAACTG	58	258
NAP1L5	CCTACTCGCCAAGATCCAAGAGC	TCTTGGCGTCATCAGGCATCTCG	58	200
TAB2	TCACAGCCTGGTCCCTGGACTAC	GCTGGAGGTTCGAGGTCCAGAAG	60	300
β-actin	TCATCACCATTGGCAATGAG	CACTGTGTTGGCGTACAGGT	55	155

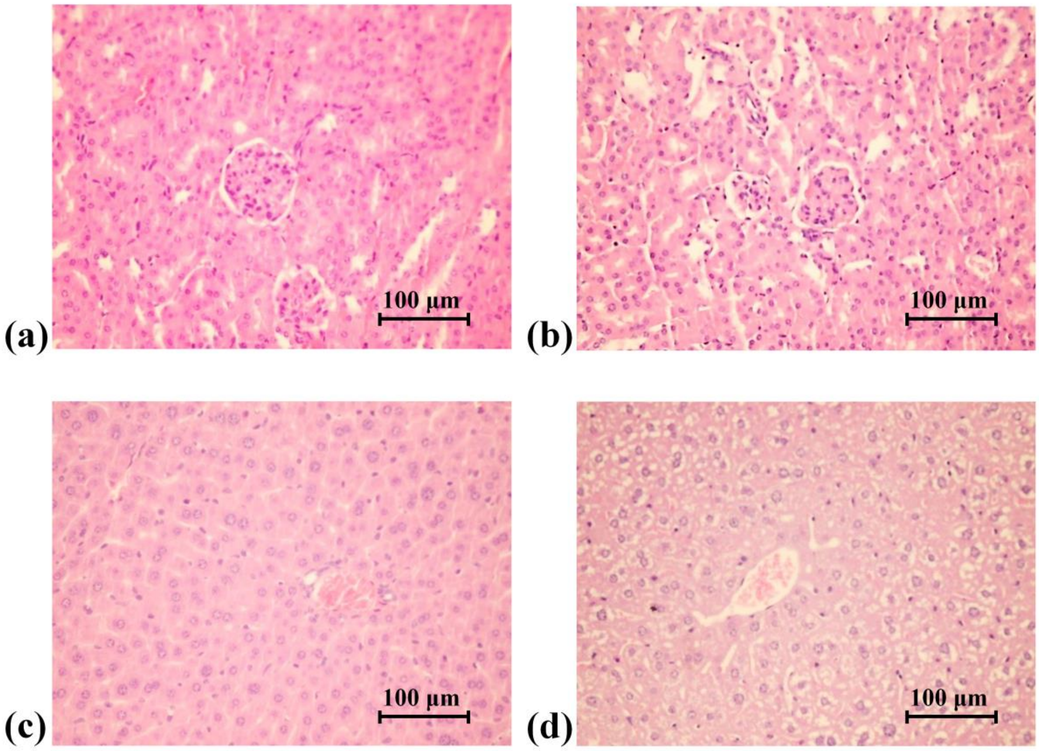

Supplemental Figure 1. Histologic findings in kidney and liver tissues following nickel administration. After oral administration of ddH_2O (control) or 100 mg $NiCl_2$/kg/day for 15 days, the mice were inoculated with or without BEAS-2B cells (1.5×10^6 cells/mouse) via intravenous route. The mice were continually exposed to ddH_2O or $NiCl_2$ for 60 days and sacrificed at 75 days. The damage in kidney and liver tissues were investigated by H&E satin. (a and c) Section of kidney and liver from ddH_2O group mice. (b and d) Section of kidney and liver from nickel-treated mice. Photographed at 100× magnification.

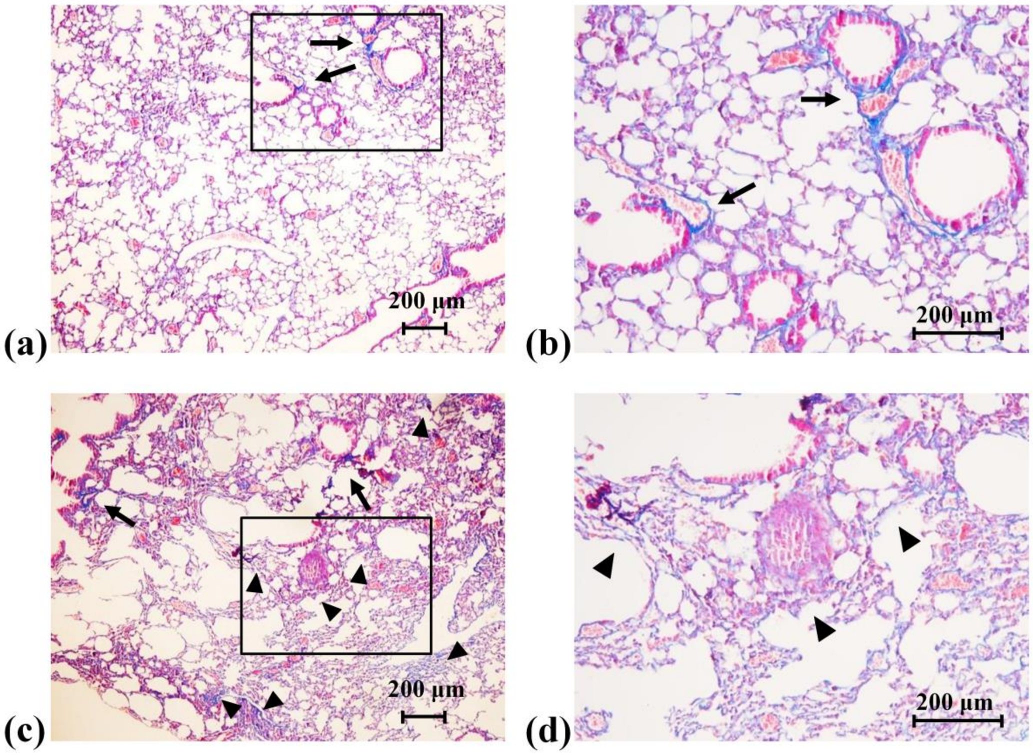

Supplemental Figure 2. Images of Masson's trichrome staining of lung tissue. After oral administration of (a and b) ddH_2O or (c and d) 20 mg $NiCl_2$/kg/day for 15 days, the mice were inoculated with BEAS-2B cells (1.5×10^6 cells/mouse) via intravenous route. The mice were continually exposed to ddH_2O or $NiCl_2$ for 60 days and sacrificed at 75 days. The compositional range enclosed by the quadrilateral of (a) and (c) are enlarged in (b) and (d), respectively. The black arrows point to the tracheal muscle, the black triangles point to the colonization of cells and the square shows the high magnification. Photographed at 40× and 100× magnification, respectively.
